# Eliminating Drawbacks to Success in Dental Practice*Dr. F. W. Sage, the author of this composition, is well versed in the complicity of the dangers that beset the practice of dentistry. He was in practice many years and recently retired to devote his remaining years to a peaceful contemplation of his success. Dr. Sage writes from a life experience and keeps up the interest with his timely wit.—*Votre Ami Dentaire*.

**Published:** 1923-02

**Authors:** Frank W. Sage

**Affiliations:** Cincinnati, O.


### ELIMINATING DRAWBACKS TO SUCCESS IN DENTAL PRACTICE *

FRANK W. SAGE, D. D. S., CINCINNATI, O.

Most of us dentists realize, on looking back to our early entrance into the profession, how limited was our vision over the far-reaching field opening before us. As matriculants in the dental college how confident were we. Our two or three years under a preceptor, assisting him in his laboratory or at his chair, led some of us no doubt to feel that the college course was to be for us individually, merely a finishing off with various ornamental flourishes, of our already acquired knowledge. All our preceptor knew we believed he had imparted to us, and we esteemed him the "best ever" among preceptors. Was he not an active member both of the state and the national dental associations, and had he not said in so many words that two years in a dental office was worth four in most dental colleges? On the whole we were feeling rather indulgent toward the college proposing to become to us an *alma mater*.

At the "quiz" we occasionally ventured to call in question some statement of the lecturer. The gentleman took this good-naturedly, and some of our classmates who kept rather in the background, were visibly impressed by our show of superior knowledge. Metaphorically they removed their hats ever after, in our presence. It was all rather flattering, and we were feeling quite well satisfied with ourselves.

It was in the clinics that we trimmed our lights for our most brilliant display. Our demonstrator one day announces an unusually difficult case presenting that

* Dr. F. W. Sage, the author of this composition, is well versed in the complicity of the dangers that beset the practice of dentistry. He was in practice many years and recently retired to devote his remaining years to a peaceful contemplation of his success. Dr. Sage writes from a life experience and keeps up the interest with his timely wit.—*Votre Ami Dentaire.*

afternoon for operation, one calculated to tax the skill of any but the most proficient. Who will undertake it? He smiles around the circle of students one after another examining the patient in the chair. Others hesitate; we decide to undertake it. Bravely we roll up our sleeves. We will show these classmates, whose preceptors we feel assured, could not measure up shoulder-high to ours.

Awhile all goes well. Crowding about the chair our classmates get an occasional peep in, and glow admiringly. Occasionally we stop to explain just why some less expert operators fail at this point, lose sight of the finer requirement of the case just there.

But now perplexities begin to trouble our innermost self. The obvious difficulties have been triumphantly overcome, but what mean these other unforseen difficulties lurking behind the outer circle of impediments? Still we press on, a trifle irresolutely, secretly a bit daunted, feeling that our best efforts are being mocked by jealous fate. To call upon the demonstrator of the clinic for a helpful suggestion we feel would be an unbearable humiliation.

Now one of the humbler sort among our classmates ventures a suggestion. What presumption! We heed him not. We stop to wipe our perspiring brow.

Ah, here uninvited comes the clinical instructor. He pushes through the circle, glances at our work, offers a brief suggestion, is off to the next chair. The "humbler sort" smiles; the suggestion is a repetition of his own modestly offered a moment ago.

Can this be possible? Our instructor, not half as old as our preceptor, has told us something of which our preceptor must have been ignorant. This is our very first intimation that the old man was not the possessor of all dental knowledge.

We finish the operation, but our crown has tilted, sits awry on our brow. Alas for human frailty! Poor humbled self-sufficiency!

Had we only known dentists of widest repute fail at times. At the meeting of the American Dental Association, many years ago, a dentist of national and international renown as a gold operator, failed at a clinic to make a perfect gold filling, although every facility was offered him. Another, equally renowned, performed some service which provoked a looker-on to say later, "We have hundreds of boys in Ohio who could do better than that." He stood face to face with the disconcerted operator, as he spoke. (Searchlight breath.)

Notwithstanding appearances these dentists must have operated under disadvantages. Take any dentist away from his own operating equipments and he is at once at a disadvantage. No matter how well supplied with every requisite the drawers and shelves of the cabinet at his elbow may be, all is unfamiliar. He can not, as in his own office, reach out and lay his hand on a needed instrument almost without looking. This is a real handicap.

Under circumstances most favorable the average dentist, if not the others far above the average, spends more or less time doing over operations not done quite right in the first instance. Let any dentist go the rounds of offices in his own town or city, or better still away from home in the offices of dentists not familiar friends; let him note the cases of doing over to get matters right that come to this dentist, and he will find something to set him seriously to thinking. In not a few instances he must be impelled to exclaim, "How can this dentist be making any money, with this aftermath of unsatisfactory services pressing upon him?"

Just to test the matter come with me. We will go the rounds of dental offices, look in for an hour or two, if time *and* dentists permit. Ah, here in Dr. Bizibee's office we hit something, first thing.

A lady comes in who complains that she can not use her false teeth. Our friend the dentist, invites us to examine the case. The full denture at a glance, seems all right, color, articulation, arrangement, fair at least. Any one but a critically disposed dentist would say so. We remove the plates, take a keen look at them, likewise at the patient's mouth.

It is one of those in which after extraction, almost no absorption of the ridges has taken place. The upper plate has a full rubber gum all around. This has been made quite thin, to overcome fullness. Looks not too full. However, she complains that it pushes out her lip too much. The arrangement of the teeth we do not like. Too much of a horseshoe rounding out. The front teeth, no matter how her own teeth were set, must be set further back and straighter across as to the six anterior. Then from the cuspids back they should be set further in, and nearer in a straight line back. (Old stuff you say. True, yet dentists years and years in practice, fail to observe the requirement. Seem not to understand that you must, in some cases all but ignore the ridge, in setting the teeth.)

These plates had been thrice made over. The lower six teeth had to be inclined forward at an angle of nearly forty-five degrees, to come near to an articulation with the upper. No wonder they tipped when she attempted to bite. Must be made over once more. We must proceed along an entirely different line. This is all wrong.

Suggestion: Open the bite to allow of setting lower teeth well in. They must go up higher upon the ridge, must stand further back, or she will never so balance the denture as to eat on it. Sometimes the second lower molars will be found, under this rearrangement, seemingly tipping too far inside the ridge. Never mind that. There is such a thing as regarding too carefully an arrangement imitating the arrangement of the natural teeth. In many cases that consideration needs to be all but ignored.

As to the upper plate; omit the front rim. Six front teeth may set on the gum. Some dentists seem to think this applies only to temporary sets. Hard ridge all around, you object? Plate would never stick. Oh yes it will. This brings us to impressions.

They tell us that Dr. Hall, who introduced his black wax for getting a trimmed impression tray, did not, when giving a clinic, take even a second plaster-film impression. Well, he may not have needed to. But if your first trial with the plaster film fails to give you an impression that "sticks," try a second plaster film, try still again, until it responds to suction. For convenience in deciding certainly that the last thin coating of plaster has covered the entire surface of the impression, I paint lightly with any coloring compound. (Not varnish, of course not.)

I do not claim expert knowledge, but I have made plates for hard-roofed mouths, using this method for the impression, which despite the omission of the front rim, in upper sets, held very firmly. If, however, you fail to get adhesion of the impression by the Hall method, try the method described by Dr. Prothero, or perhaps it is Green, or Supplee, I have forgotten. The best impression compound is used, taking the impression, trimming away surplus, wiping dry, warming over spirit lamp, dipping into fairly hot water, reapplying. Repeat a second, a third time, until adhesion begins to take place. I have made plates upon models thus obtained, that stuck as if glued, the patient in one instance returning to me to remove the plate for her. This too, when no front rim was used.

Plates so snug fitting do, however, make the tissues sore, before long. One lady declined my offer to paint with iodine her slightly inflamed palate. Did not feel sore, and she was too delighted to have a plate that stuck, to reason further.

Do not be afraid to omit the front rim.

(Note—On my suggestions above detailed, the dentist who had made the lady's teeth over a third time, tried again, and reported that they were all right; lady could eat with them. Well pleased.)

With our improved base-plate materials it is sometimes of advantage in full dentures, or in either full upper or lower, to make two or three carefully fitted and trimmed sets of baseplates with teeth articulated and mounted with stiff wax. Try these experimentally in the mouth. Ofttimes the patient will express a preference. He will also later be more likely to feel reassured, if you tell him the difficulties he is experiencing will pass away. Less likely will he be to complain as I heard one lady complain not long ago, whispering to a friend with her, "He knows it isn't right, and thinks only of getting rid of me."

It is of advantage to you in another way, this extra precaution above recommended if it causes your patient to say, "Dr. Blank never took such pains with me as you are taking."

Early reputation-building this might be called. You widen the circle of confiding patrons. Well worth while, isn't it?

Any old cheap teeth will do for these trial sets. May be used again and again. What an advantage it would be if somebody would invent a base plate so strong and an equally strong wax attachment, that our patients could wear the sets a few days, by way of testing them.

Read recently, that it is better to take impression by the Hall method, with the mouth open, for lower full sets. Who knows as to that? I made a full set for a woman years ago, who did not wear the lower, although it stuck like a leech, *when the mouth was wide open.* An accident, since I took both impressions by the old method. Was not then impressed with the full significance of what was presenting.

Here we are in another dentist's office. Lady presents for consultation. Another dentist reset a pivot tooth for her, overlooking the fact that the root was split. Severe inflammation followed. Extraction of tooth, a cuspid, revealed the fact that cement had pressed through causing the inflammation.

(Note—Where a pivot crown has come out and you find the pin covered with cement, you may suspect a split root. Cement-coated pins do not easily come out. At a glance the root may seem all right. But a little lateral prying reveals the break.)

The dentist in whose office we now are, had made a bridge supported at the distal end by an inlay in the first bicuspid and forward by another in the lingual surface of the central incisor. The bridge carried a lateral incisor besides the cuspid. After less than a month's wearing, the bridge, when the lady presented, was loose.

Ill-judged operation. Fine jeweler's work, poor dentistry. No inlay in a central incisor need be expected to withstand the strain of a bridge of this length. The utmost that need be expected of such an inlay in a central, is that it shall support a single lateral swung in. An inlay reinforced by three pins, according to the latest improved method recommended by Dr. Hinman, usually proves durable.

An old rule ignored in this instance, is to avoid carrying a bridge from behind around the anterior curve of the mouth, unless very strong support distally is assured. Even then nothing less than a pivoted crown carrying the forward end, is to be depended on. An inlay will almost invariably loosen, under the strain.

Now here we are in Dr. Nearcorrect's office. Man in his chair complaining of bicuspid being sore to touch. We catch an occasional remark, * * * "It hurt when you filled the root, hurt all the time you were working at it. Kept me awake all that night." The dentist smiles indulgently. "My dear sir, they often act just that way. A little painting of the gum will make this all right. Forget it, forget it."

All wrong, say we. A properly treated root never hurts while being filled. Properly treated means repeatedly treated, then treated some more, until it may be probed to the apex without sensation. Good thing that less of this is now being done. The root that is still foul after two or three disinfectings, needs a forceps, not a double extra fine bristle. Strange how many good mechanics in dentistry haven't the first idea of dental therapeutics. And vice versa.

This dentist simply did not know.

Hallo, here comes another. Bridge too high. Can not shut teeth together on opposite side. "Oh Doctor, you're surely not going to grind it some more!" the lady exclaims. But he certainly is. Grind and grind *and* grind until—until what? Until the white cement begins to show. Now it is at last seemingly right. But that exposed cement? "Oh bother the cement! I've had enough trouble with the confounded bridge!" we imagine him saying, as he swings the lady out of his chair.

One of the most perilous features about bridge making is not in the making but in the setting. (Fresh "bull," from Ireland.) Hot summer day. Cooled glass slab and gradual, careful mixing, all in vain. Your bridge sticks when five-sixths of the way to place, and—there you are. Frantically you pry and gouge to get it off,—in vain.

Dr. Richmond, when he introduced his all gold crown, going from office to office, directed that a hole be made to allow of the cement escaping. A long-forgotten admonition. Excellent one to recall and use, when setting a bridge on a hot day. Try it. Some little trouble to fill the vent-hole, but not as much as to grind later on, and ruin your bridge. Half a day wasted in grinding.

I have sometimes temporarily set bridges with chlora percha.

Ah, here enters another dissatisfied one. She is telling her companion all about it, and four other waiting patients are taking it all in. Almost as good as a movie-show they seem to regard it. Incautiously the dentist enters to speak to another waiting one. Instantly the last-comer pounces upon him. He must hear her first; she is in a great hurry. Tooth under that big inlay ached all last night. She will be back in half an hour and he must be ready to attend to her at once. As she goes out tossing her head, the dentist invites us into his operating room. We are alone. He knows we overheard. Wants to explain. Perspires.

Lady something of a crank. Easily hurt. Said tooth had ached, but you know you need pay no attention to such statements in such cases. (!) Well yes, she did wince when warm air from the syringe was used. Five minutes later declared it was still aching. No near pulp exposure. Went right ahead and set the inlay.

Responsibility on this dentist? Surely so. "In case of doubt choose the safe course." But he had made the inlay, had saved time for her, and—confound it, what was the matter with her tooth, anyway? (Probable near-exposure of abnormally developed horn of pulp.)

This dentist should have heeded the first red flag. Gutta percha filling, and—wait awhile. No gold inlay at present. Fee gone glimmering, but later trouble avoided. No adverse advertising to a roomful of waiting patients. Forceps as a finale, but no just ground for complaint against her dentist. Seemed very cautious; no fault of his, she tells her friends. (Query: is she a crank after all?)

Enough of this. We should like to add a few pages showing what share of blame properly rests on the patient's shoulders for failure of dental operations. But that is another long story.

So many dentists lack poise, go off half-cocked. "This case is easy enough, just like the other I had last week," they will say after a mere glance. Mark the statement; the dentist who takes a lot of time peering into a patient's mouth, using his mouth mirror here and there, searching back and forth and across with his exploring points, going all over the teeth again and again, is the dentist who is least liable to fall into some hidden trap. He will not fill a large cavity in a molar, or insert an inlay, only to discover later a much larger cavity craftily concealing itself beneath the gum, too large for filling, in the same tooth. He will not plunge ahead without much searching inquiry as to difficulties liable to be encountered, in this mouth requiring a full denture. He may need to talk a patient out of a lot of preconceived notions, before taking that impression, may have to suggest to him or her the expediency of his or her looking further for a dentist, if symptoms of hopeless crankiness appear. Many dentists dare not go to such a length. Yet what is the alternative? Go ahead and get yourself involved in endless difficulties of the patient's imagining? By no means. If you find you have lost a patient console yourself by thinking you have likewise escaped a lot of annoyance. If word of it gets abroad is that likely to injure you? Far from it; people whose opinion is worth while, will rather commend your action. (Think how that other dentist is suffering.) It will act as a corrective of a widespread feeling that dentists can not afford to let the smallest fish escape their net. Do bankers and business men and lawyers, allow people to dictate as so many dentists do? Talk about elevating the profession. What the profession needs is to elevate itself by a more conspicuous manifestation of self-respect.

In all the foregoing we have referred to errors of the dentist which the patient has discovered. Other errors the dentist blunders into of which he alone has secret knowledge. That is another story of which we may particularize later.

Unquestionably some patients are captious, from the first hold themselves in an attitude of rather seeking occasion for complaint. Such are cranks about many other matters. If in changing from a light summer shoe they find an hour's discomfort in the heavy, high-topped winter footgear, they would enjoy hauling the shoe manufacturer over the coals, could they get at him. I have had patients return to my office complaining about the discomfort of teeth I have made for them; then months afterward on questioning not the complainant but say for instance, her sister, have been assured that she wears the set with perfect satisfaction and comfort, never having had any alteration made. The wife of a judge stopped me on the street one day, telling me the set I made for her was wholly unsatisfactory, hinting darkly that she might try another dentist. She made no reply to my suggestion that she return to my office and let me see what was wrong. She did not do so.

Fully a dozen years later I met her in a store. She spoke to me first, I not recognizing her because of gray hair and other changes wrought by years. Said she would like to have me make her another set of teeth. I found she had been wearing my first set all those years. On my reminding her of her threat to see another dentist, I saw she had forgotten all about that. She flushed scarlet, apologized.

We dentists are peculiarly exposed to criticism, often grossly unjust, undeserved criticism. Much of this might be avoided if only we would take more pains to impress our patients with an appearance of earnestness and sincerity in our every ministration to their needs. Give all necessary time to the first examination. Tell the patient frankly, that you need time to consider exactly the best procedure in the particular instance. Ask him to come again, even though he may see you are not engaged at the time. This creates confidence, protects you against hasty misjudgment in case later matters seem to have gone wrong. Quite as important; it saves from actual error, that is, if the dentist really does need to consider this operation over and against another suggesting.

It is better to lose time before going ahead, than to have to undo and begin all over. (Sounds like a childish admonition, yet many dentists no longer tyros in practice, need occasional reminding.)

In the business world salesmen, clerks behind desks, even heads of departments, have someone higher up watching their doings, calling them into conferences, admonishing, approving, correcting, all in the interests of the business. The dentist is a law unto himself, acknowledges no leadership excepting that of the dental society or convention. The progressive dentist goes away to larger towns, cities, takes note of what the advanced men in the profession are doing, pays for special instruction in new methods, goes back home resolved thereafter to do better work. Some have been suspected of keeping to themselves knowledge thus acquired, of allowing a brother dentist to blunder on in the old way, using obsolete methods. True in some instances, no doubt.

Let us wake up, study to reduce our misfits, total failures. Be earnest, go slow, establish a reputation for painstaking. Then the crank who complains of your incompetency before a roomful of waiting patients, will make little impression on them, for they know your honesty, capability, progressiveness. Then you will find you are making some money.

